# Feeding responses of the golden jackal after reduction of anthropogenic food subsidies

**DOI:** 10.1371/journal.pone.0208727

**Published:** 2018-12-07

**Authors:** József Lanszki, Matthew W. Hayward, Nikolett Nagyapáti

**Affiliations:** 1 Carnivore Ecology Research Group, Kaposvár University, Kaposvár, Hungary; 2 School of Environmental and Life Sciences, The University of Newcastle, Callaghan, NSW, Australia; University of Sydney, AUSTRALIA

## Abstract

Little is known of the resources that limit or promote the rapidly expanding golden jackal (*Canis aureus*) population in Europe. We hypothesised that in an area of intensive big game hunting, a reduction of the main food resource (human subsidised big game viscera) would result in dietary switching. We used multivariate analyses to test whether the dietary composition of 200 jackal stomachs varied between two 2-yearly survey occasions, the first without big game viscera removal (availability of 68 kg viscera/year/km^2^) followed by a period with viscera removal (minimum of 50 kg of viscera/year/km^2^ removed). The proportion of empty stomachs and the stomach wet content weight did not differ between the two periods. Even after the reduction of food subsidies, the primary food of jackals was viscera and carrion from wild ungulates (frequency of occurrence: 45% *vs*. 30%; wet weight: 55% *vs*. 29%, respectively), and scavenging was not affected by season or sex. Log-linear analysis of frequency data revealed no significant differences between survey occasions in consumption of either food type. MANCOVA of wet weight data revealed that in the first period with food subsidies jackals consumed a higher proportion of adult wild boar (11.6% *vs*. 1.3%; from predation or scavenging), while juvenile wild boar (0 *vs*. 11.8%; from predation or scavenging), domestic animals (0.8% *vs*. 6.2%; mostly from scavenging) and invertebrates (2.6% *vs*. 4.1%) increased in the second period. The stomachs in the second survey occasion contained more varied food items, but the trophic niche was not significantly wider. The feeding responses of this mesopredator to the reduction of food subsidies were less pronounced than expected. Because in high big game density areas, wild ungulate carrion from different mortality causes are available in high quantities throughout the year, predator populations can be maintained despite the high amount of viscera removal.

## Introduction

The range expansion and population increase of some mesopredators (relative position of ‘medium-sized’ predators within food webs [1] that can adapt to both natural and human-dominated environments has been regionally or globally observed [1–3]. The ‘mesopredator release effect’–where mesopredators increase when apex predators are removed [4]–might be facilitated by the easy access and large amount of anthropogenic food resources available [5–6]. For these animals directly or indirectly derived food resources [7] near settlements are mostly livestock, carrion of domestic animals and garbage [8–9], while farther from settlements these are mostly viscera of big game (wild ungulates) left behind by hunters and wildlife managers on the area (indirectly derived anthropogenic food resource) and carrion of big game from different mortality causes [7, 10–11].

Large predators provide important regulating ecosystem services (e.g. biological control via top-down regulation of pest animal populations; [12–13]), however the ecosystem function of mesopredators can also be measured by their economic and ecological role [4, 14]. Mesopredators can have a substantial role in sanitation around settlements by the removal of waste and carcasses [15–16]. Furthermore, by limiting the abundance of smaller predators, mesopredators may have an indirect positive effect on biodiversity [4, 14] through controlling mammalian pests [14, 17]. Overall, the functional roles of mesopredators in regulating trophic cascades can have a significant impact on the ecosystem [4, 14].

In human-dominated environments, ecosystem dis-services associated with high mesopredator densities are better known, including as vectors of diseases [18], as predators of wild ungulates and of domestic animals in pastoral zones [1, 9, 19]. However, the consumption of an animal does not necessarily mean that it came from predation, e.g. in the case of scavenging. Ungulate density has a direct influence on carcass feeding [10] and open garbage dumps are attractive to mesopredators and may cause unnaturally large aggregations [5, 8, 20]. The management of certain common generalist carnivores with high population densities may be necessary by non-lethal methods or lethal removal [16, 19]. One possible method of controlling overabundant carnivores may be a drastic reduction of anthropogenic food sources. It was found [5] that removing anthropogenic food increased home range size and decreased survival of red foxes (*Vulpes vulpes*). Similarly, others [21] found that golden jackal (*Canis aureus*) survival under food reduction decreased and was lowest for dispersing individuals. Experimentally confirmed [22] that human-resource subsidies alter the dietary preferences of dingoes (*Canis lupus dingo*). Medium-sized canids may respond to changing amounts of food by dietary switching [23–25]; jackals use of both prey and directly or indirectly derived anthropogenic food sources. They may also respond with changes in body size [26].

The range and abundance of the golden jackal (hereafter jackal) has been rapidly increasing throughout Europe [27–28]. The population growth and expansion of jackals might be facilitated by several factors, including flexible social behaviour [8, 29–30], varied dispersal patterns [21, 31], legal protection (e.g. in Bulgaria in 60’ [32]), the scarcity of larger competitors [28, 33], poor population management [32], abundant food resources [9, 11, 15, 17], poor sanitation conditions around settlements [6], transformation of habitats (e.g. land use changes, intensification of agricultural production; [34–35], global climate change (e.g. range shift; [28]), but there is no consensus of what is driving this rapid range expansion [28, 33].

We hypothesise that in an area of intensive big game hunting with high jackal density, reducing the primary food subsidy (big game viscera; [11]) will result in pronounced food switching. To test this hypothesis, we manipulated food subsidies at a landscape scale over four years in the first manipulative experimental test of the role of anthropogenic food subsidies on jackal diet. Our predictions were that this would lead to (1) reduced stomach content weight and body mass of jackals, and (2), an increase in the consumption of food types acquired by depredation by jackals, such as (a) small mammals and/or (b) big game carcasses and/or big games (adult and/or young individuals) as prey. Furthermore the consumption of suboptimal food types (with low energy values), such as (c) plants and/or (d) garbage would also increase. This is because jackals in Hungarian agroecosystems primarily eat small mammals, and wild boar (*Sus scrofa*) after severe winters [17]. Considerable calf predation occurs in small enclosed areas in India where golden jackals at high density also kill chital (*Axis axis*) calves and persist on plants [36]. In Serbia, jackals consume easily available waste (e.g. domestic animal carrion) from dumps [15].

## Material and methods

### Study area

The 165 km^2^ unfenced study area is located in the Pannonian biogeographical region of SW Hungary (Lábod region; centre: 46°11’ N, 17°30’ E, S1 Fig). This is a flat, lowland area with sand-dunes (125–190 m, above sea level). Forestry, wildlife management and crop cultivation are the predominant land use of the region. The vegetation consists of forests (53.5% of all land) of English oak (*Quercus robur*) (31.5% of forested areas), willow (*Salix* sp.), as well as alder (*Alnus* sp.), linden (*Tilia* sp.) and black locusts (*Robinia pseudo-acacia*). The age of the forest is under 40 years. In the arable areas (36.7%), row crops, oilseed rape and cereals dominate, but pastures (7.5%), ponds and wetlands (1.1%), human settlements and orchards (1.2%) also occur [11]. Within the study area or directly around it there are nine small villages, with less than 2,000 inhabitants per settlement. Human population density is 8.2 individuals/km^2^. The climate is continental with some sub-Mediterranean features (e.g. moderately wet and relatively mild winter). During the study period, the mean (± SE) annual temperature was 11.1 ± 0.2°C, the average number of frost days was 81.6 ± 10.0 days, the annual number of days with snow cover was 16.6 ± 5.2, average snow depth was 20.5 ± 0.4 mm, and mean annual precipitation was 712 ± 104 mm (S1 Table).

### Study species

Intensive big game management via trophy hunting of fallow deer (*Cervus dama*), red deer (*Cervus elaphus*) and wild boar occurs in the study area, while roe deer (*Capreolus capreolus*) is a less important hunted species. The legal hunting seasons of the fallow deer is October-February, the red deer is September-January, the wild boar is year-round, and the roe deer is year-round except March and first half of April. In the 482 km^2^ hunting area of the Lábod district (SEFAG Co.), the mean (± SE) annual harvest densities (hunting bag) were 2.12 ± 0.39 fallow deer/km^2^, 1.10 ± 0.07 red deer/km^2^, 2.01 ± 0.24 wild boar/km^2^ and 0.30 ± 0.03 roe deer/km^2^ during the study period (S2 Table). Small game species, such as pheasant (*Phasianus colchicus*) and European brown hare (*Lepus europaeus*), were rarely hunted (hunting bag < 0.1 individuals/km^2^). There are additional feeding in the area for wild ungulates. There are no accurate population estimates for these species.

Data on individual body mass data and hunting bag sizes for all the big game species of the area were used to determine the minimum quantity of big game viscera (some of which is destroyed, but a substantial amount of which remains at the site of harvesting) resulting from human hunting activity. We calculated the viscera (stomach, intestines, oesophagus, heart, lung and liver) weight with a constant factor of 25% compared to full body mass [37] in both survey occasions from the weight of field-dressed animals (i.e., with viscera and blood removed). In the second survey occasion, viscera was collected and deposited by professional hunters in a properly fenced location inaccessible to jackals.

Between January 2012 and November 2013 (the first survey occasion when there was no viscera removal [11]), and between December 2013 and October 2015 (the second survey occasion when food subsidies were experimentally manipulated via viscera removal), the number of harvested big game was 1903 and 1526 individuals in the study area, respectively. Of these, 1821 and 1408 animals were shot during hunting activities, 1789 and 1341 individuals (98.2% and 95.2%) of which had body mass data collected in each survey occasion, respectively. In addition, 82 and 88 game individuals were found as carrion (mortality resulting from wounding and the loss of the individual, poaching, and some non-hunting related mortality, e.g. road casualties, diseases), and we had body mass data for 23 and 18 of these (28.0% and 20.4%) in the two survey occasions (these were not removed from the area). Estimation of this carrion was based on the number of registered individuals and known average body mass data by species, sex and age group separately as detailed elsewhere [11].

There are two sheep (merino) farms in the area (separated by 17–18 km). Sheep of the Homokszentgyörgy flock graze outdoors all year round, but are kept in a barn overnight. The Nagykorpád flock is kept outdoors in summer and autumn, but there is no barn and they are in the open during the night. One shepherd and a few sheepdogs (smaller sized herding dogs) accompany each flock. Domestic ungulates are registered and marked individually, and dead animals are compulsorily dispatched and disposed. Live animals are sold, therefore slaughtering can occur very infrequently, whereupon viscera are available for scavengers.

The mean (± SE) jackal density of the area was 0.27 ± 0.02 groups/km^2^ plus 0.05 ± 0.01 individuals/km^2^, (0.31 ± 0.01 groups/km^2^ plus 0.04 ± 0.03 individuals/km^2^ and 0.25 ± 0.04 groups/km^2^ plus 0.07 ± 0.02 individuals/km^2^ for the two 2-yearly survey occasions, respectively) calculated from records of seven surveys between March 2013 and November 2015 by the stimulated calling method [38]. Jackal groups and individuals (when a call response from a single jackal occurred) were treated separately. The mean (± SE) annual hunting bag density of the jackal was 0.19 ± 0.04 individuals/km^2^, while that of the red fox was 0.11 ± 0.01 individuals/km^2^ (S2 Table). In Hungary, unlimited hunting is allowed for both jackal and fox. There are no grey wolves (*Canis lupus*) in the area. No specific permissions were required for the study.

### Sample analysis

We investigated the feeding habits of jackals by analysing stomach contents from samples provided through legal hunting with sample sizes of n = 62 and 138 in the first and second survey occasions, respectively. We measured the body mass of jackals to within 0.1 kg, then stomach samples of jackals were removed and stored at –18°C prior to analysis. After weighing the stomach content separately for each food type, food items were analysed both macroscopically and by microscope on the basis of hair, feather, skin, bone, dentition and chitin shell characteristics using standard procedures [39]. Occasionally, in cases of more advanced stages of digestion and when small food items were difficult to count and identify, the stomach contents were washed through a 0.5 mm sieve and then all recognisable prey and food remains were separated.

To calculate diet composition, we took into account the minimum number of food items that could be identified in the stomachs. We determined the percentage composition of food items in the stomach samples on the basis of 1) relative frequency of occurrence (RFO; proportion of the total number of occurrences of all items in the sample), 2) frequency of occurrence (FO; proportion of stomachs containing a given food item) and 3) wet weight (measured at an accuracy of 0.01 g) of all individual food remains found and separated in the samples (W; proportion of a given food item wet weight in the total wet weight of food remains found in the stomachs).

The following 16 major food types (supplemented by three categories used by [11]) were used in the comparative analysis of diet compositions: 1 –viscera and ‘other carrion’ of wild ungulates [i.e. all remains left by hunters including internal organs with the contents of the digestive system, skin remains, ends of cervid legs and heads of non-trophy females. In addition, old or fresh carcass or remains of carcass, dead before being taken by a jackal, e.g. which can appear with signs of poaching or decomposition], 2 –adult wild boar, 3 –juvenile wild boar (piglets and hoggets), 4 –adult deer (red deer or fallow deer), 5 –juvenile deer, 6 –adult roe deer, 7 –carnivores (wild), 8 –small mammals, 9 –European brown hare, 10 –domestic animals, 11 –birds (wild), 12 –reptiles and amphibians, 13 –fish, 14 –invertebrates, 15 –plants (from direct consumption), 16 –inorganic materials. The occurrence of viscera and ‘other carrion’ in stomachs indicated human hunting or poaching, and these subcategories were taken together, as it is often difficult to distinguish between them. Fly larvae or pupa in the stomach content indicated feeding on carrion, but jackals might have been feeding on injured or dead ungulates overnight [17], and, in these cases, larvae were missing. Adult wild boar and adult cervids were separated from the viscera and ‘other carrion’ category, because contrary to the first category, predation could not be excluded in these cases, although, in the case of healthy individuals, there is a low probability of this [11]. In these cases, predation and scavenging are also possible. As with sheep, no cow or poultry losses reported attributed to jackals, therefore consumption of these domestic animals mostly might came from scavenging.

We categorised the jackals examined according to sex and season, i.e. 1 –December—April (winter and early spring, mating and gestation period of jackals, and gestation period of cervids), 2 –May—July (spring end and early summer; pupping of jackals, calving of cervids and early parental care period), 3 –August—November (teaching young jackals for hunting, and intensive trophy hunting of cervids) [40–41].

### Data analysis

Analysis of covariance (ANCOVA, GLM procedure, SPSS 11.5) was used to compare the estimated total mass of detected mortality from human hunting and other mortality causes (as dependent variable; kg/km^2^) found between the two 2-yearly survey occasions (as fixed factors) depending on season (as covariate; three seasons). Three-way ANOVA (Bonferroni post hoc test) was applied in the adult age group category of jackals to examine body mass (after logarithmic transformation of the data) differences between the survey occasion, season and sex.

The chi-square (χ^2^) test was used for distribution analysis of the empty and non-empty stomachs between the two survey occasions. For non-empty stomachs, we assessed the effects of food subsidy manipulation (survey occasion), season and sex after logarithmic transformation of the data for stomach content weight with ANCOVA (with body mass as covariate).

Because, relationships between basic data of the three calculation methods (RFO, FO and W) were significant according to the 16 main food taxa (Spearman’s rank correlation, four years, n = 96, RFO–FO: r_S_ = 0.992, P < 0.001, RFO–W: r_S_ = 0.891, P < 0.001 and FO–W: r_S_ = 0.902, P < 0.001), subsequent statistical analyses were performed mainly on FO and W values. General log-linear analysis was used on FO data to test for dietary differences between survey occasion, season and sex. The unit of analysis was jackal stomach and the response variable was the presence/absence of the food item considered. The model was fitted using survey occasion, season and sex as categories. Owing to the large number of comparisons (16 food categories), we adjusted the level of significance to 0.0031 with a Bonferroni correction. MANCOVA was applied to test differences in quantitative composition of the diet (arcsin transformed %W values as dependent variables, survey occasion and season as fixed factors and sex as a covariate. The statistical relationship between ungulate viscera and carrion availability (estimated biomass, kg/km^2^) and consumed mass of ungulates (g/jackal stomach) was estimated by a linear regression model.

Trophic niche breadth from RFO data was calculated in accordance with standardized Levins index (B_A_, rating from 0 to 1; [42]). The B_A_ values between the two survey occasions (and taking into account the seasons) were compared with a paired samples t-test. The difference between the numbers of food items per stomach between survey occasions was compared with an independent samples t-test. A minimum probability level of P < 0.05 was accepted in all statistical tests, except log-linear analysis.

## Results

### Quantity of big game viscera and available carrion

**Fig 1:**
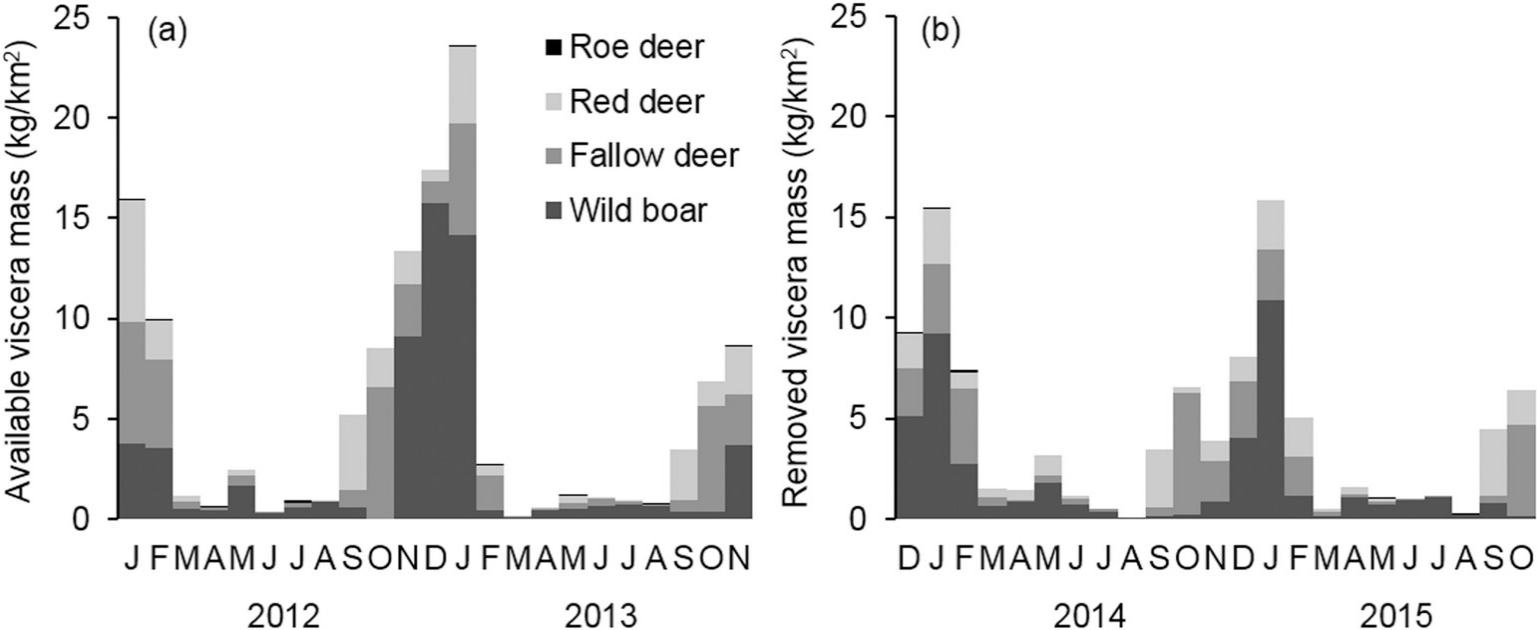
Estimated quantity of big game viscera in the study area presented by month. Left figure (a) shows available big game viscera mass for jackals, while right figure (b) shows removed viscera mass (non available for jackals). Quantity of big game viscera was calculated from the size of hunting bags of each big game species (number of individuals) and their individual body mass (kg) with a correction factor of 25% visceral weight [37]. Note, we cannot estimate the mass of viscera available to jackals from December 2013 because we removed all viscera that we were able to detect.

**Fig 2:**
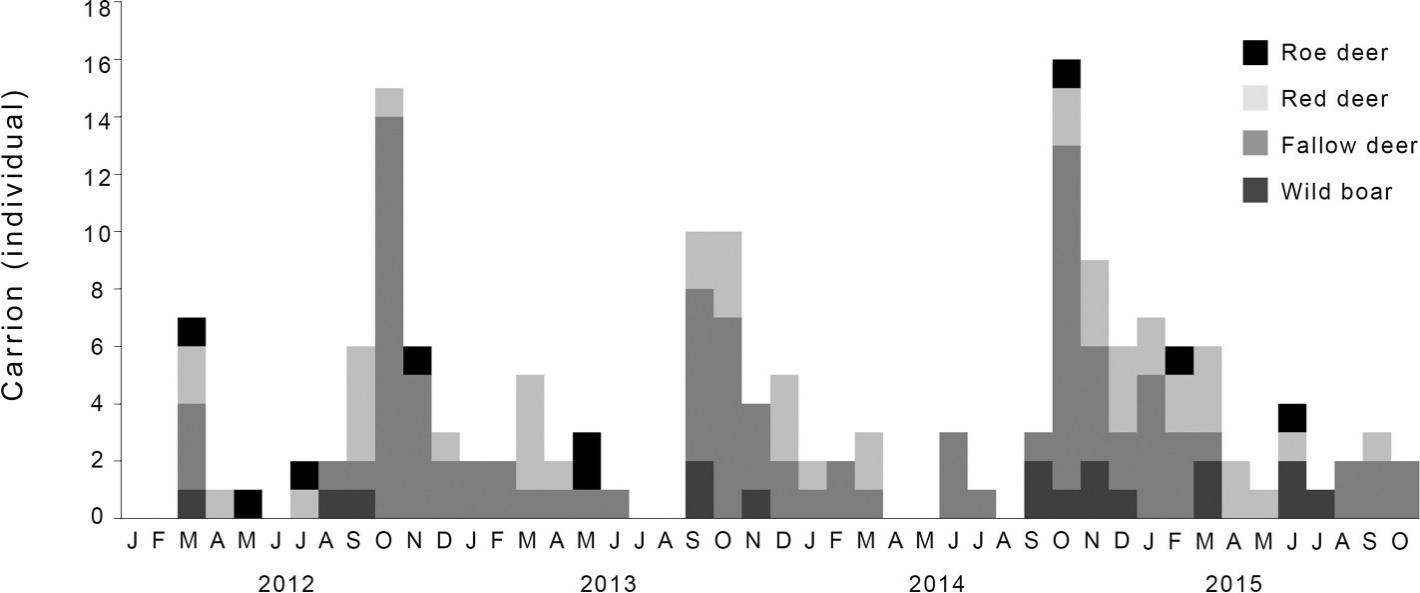
Number of detected dead big game (mortality resulting from wounding and other non-hunting related mortality) in the study area presented by month.

The total field-dressed mass of harvested big game was 271.6 kg year/km^2^ in survey occasion 1 and 198.6 kg year/km^2^ in survey 2. According to hunting bag data, wild boar was the most harvested species (47.3% and 44.2%, respectively) in both survey occasions, followed by red deer (30.8% and 23.0%) and fallow deer (21.6% and 32.5%), while the proportion of roe deer was low (0.3% in both periods). The quantity of viscera (total weight of viscera: 67.9 kg year/km^2^ and 49.6 kg year/km^2^, respectively in the two survey occasions) showed a characteristic pattern, influenced by the hunting seasons (Fig 1). Most of the viscera arose between September and February in period 1, but this was absent in the second survey occasion due to our experimental removal from the available food supply. Nonetheless, big game carrions from other detected mortality causes still provide a substantial food resource for jackals in both survey occasions (Fig 2). The proportion of these carrions (from other detected mortality) in the total sample (n = 1903 and 1408 harvested big game) for each survey occasion was 4.3% and 8.4%, respectively. The estimated total mass of dead big game did not differ significantly between the first and second survey occasion (16.8 kg year/km^2^ *vs*. 16.2 kg year/km^2^, ANCOVA, F_1,9_ = 0.005, P = 0.943) and among seasons (F_1,9_ = 0.235, P = 0.639).

### Body mass

The body mass of adult jackals did not differ between the survey occasions (3-way ANOVA, F_1,152_ = 0.429, P = 0.513), but differed depending on sex (F_1,152_ = 23.208, P < 0.001) and season (F_2,152_ = 5.348, P = 0.006). Mean (± SE) body mass of males was 11.10 ± 0.17 kg (min. 8.2 kg, max. 14.8 kg, n = 80), and that of females 9.44 ± 0.11 kg, (min. 6.9 kg, max. 12.9 kg, n = 84). Jackals were heavier between December and April (10.68 ± 0.17 kg) than between May and July (9.87 ± 0.22 kg) or August and November (9.94 ± 0.21 kg). The survey occasion × sex interaction was significant (F_1,152_ = 4.705, P = 0.032); males were heavier (10.84 ± 0.29 kg *vs*. 11.23 ± 0.20 kg) and females lighter (9.59 ± 0.11 kg *vs*. 9.38 ± 0.15 kg) in the second survey occasion than the first.

### Feeding responses

The proportion of empty stomachs (9.7% *vs*. 13.0%) did not differ significantly between the two survey occasions (Chi-square test, χ^2^_1_ = 0.459, P = 0.498). The mean (± SE) weight of food in the (n = 62 and 138) jackal stomachs examined was 137.3 ± 29.2 g and 129.1 ± 16.7 g (excluding empty stomachs: 152.0 ± 31.7 g and 147.8 ± 18.5 g) in the two survey occasions, respectively. The highest stomach content weight values were 1559.9 g (15% of jackal body mass; first survey occasion, September) and 1589.6 g (12.5% of the jackal’s body mass; second survey occasion, March). The weight of different food items in jackal stomachs was not significantly different between survey occasions (ANCOVA, F_1,163_ = 0.074, P = 0.786), season (F_2,163_ = 0.092, p = 0.912) or sex (F_1,163_ = 0.431, P = 0.512). The survey occasion × season interaction was significant for the December-April period (F_2,163_ = 5.164, P = 0.007) as jackals had lower stomach content weights in the second survey occasion compared to the first.

**Fig 3:**
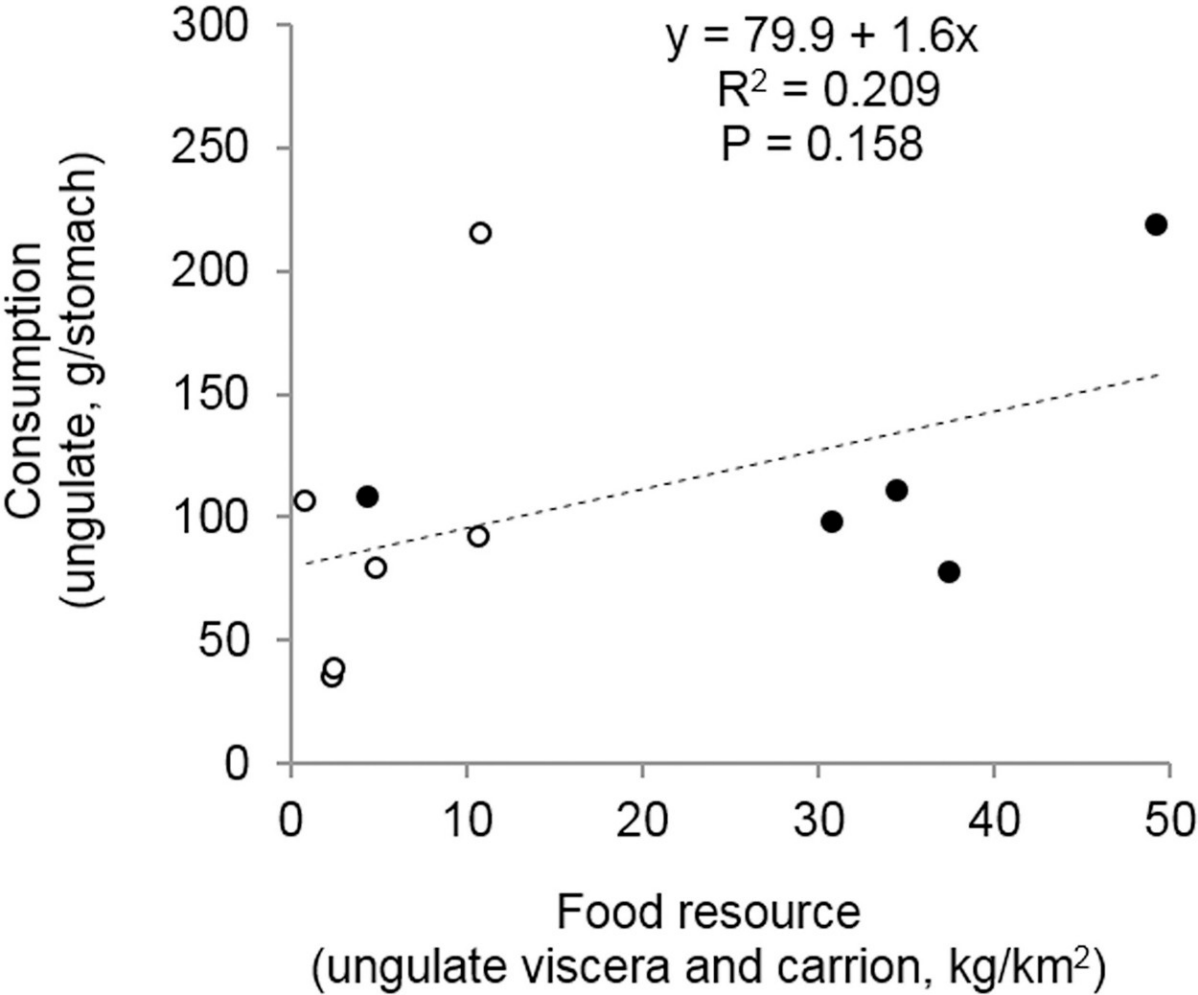
Relationship between the estimated available biomass of viscera and carrion of ungulates, and the consumed mass of ungulates. Resource estimation is based on the quantity of big game viscera in the study area (Fig 1). Carrion estimation is based on the number of known dead big game (Fig 2). Full circle–food subsidies present, empty circle–food subsidies removed (carrions are available). The dashed line indicates a non-significant linear relationship.

In the first survey occasion, when food subsidies were present, the primary food of jackals was viscera and carrion (55% of diet; Table 1). Adult wild boar was the second most important dietary component and cervids the third. In the second survey occasion, with viscera removal, the primary animal food types of jackals was also viscera and other carrion of wild ungulates, which formed nearly one-third of the diet (Table 1). Based on weight, adult cervids were the second most important and juvenile wild boars the third most important foods. Juvenile cervids (fallow deer fawns), small mammals, domestic animals (dog, poultry feather, tallow of ungulate) and plants (mainly fruits) were of similar importance (W: 6–8%); however plants were the most frequently eaten foods (FO: 38.4%). Other food types were consumed occasionally or in small amounts (Table 1). Big game consumption did not increase significantly (R^2^ = 0.209, P = 0.158) with the increase in the amounts of available viscera and carrion (Fig 3).

**Table 1:** Annual stomach content of golden jackals (*Canis aureus*) in SW Hungary (Lábod region).

Food categories	First survey occasion			Second survey occasion		
	(Food subsidies present)			(Food subsidies removed)		
	RFO	FO	W	RFO	FO	W
Viscera and other carrion	28.0	45.2	55.0	13.8	30.4	28.9
Wild boar, *Sus scrofa*, adult	7.0	11.3	11.6	4.0	8.7	1.3
Wild boar, *Sus scrofa*, juvenile				1.7	3.6	11.8
Deer*, adult	4.0	6.5	5.5	7.6	16.7	18.5
Deer*, juvenile	1.0	1.6	2.0	1.3	2.9	6.2
Roe deer, *Capreolus capreolus*, adult	4.0	6.5	6.0	0.7	1.4	1.5
Badger, *Meles meles*	1.0	1.6	6.0	0.3	0.7	0.1
Small mammals	5.0	8.1	0.9	14.5	13.8	7.2
Brown hare, *Lepus europaeus*				0.7	1.4	0.3
Domestic animals	2.0	3.2	0.8	1.6	3.6	6.2
Birds	2.0	3.2	1.1	4.0	8.7	1.4
Reptiles and amphibians	1.0	1.6	0.1	0.7	1.4	0.1
Fish	5.0	8.1	2.1	3.6	8.0	4.2
Invertebrates	15.0	16.1	2.6	17.5	21.0	4.1
Plants	24.0	29.0	6.3	25.4	38.4	7.9
Others (inorganic materials)	1.0	1.6	0.3	2.6	5.8	0.3
Number of samples (n)	62			138		
Empty from this (-n)	6			18		
Number of food items (N)	100			303		
Total weight of food remains (g)			8514			17690

RFO–percentage relative frequency of occurrence, FO–percentage frequency of occurrence, W–percentage weight of individual food remains found in the samples.

*Fallow deer (*Cervus dama*) or red deer (*Cervus elaphus*).

**Fig 4:**
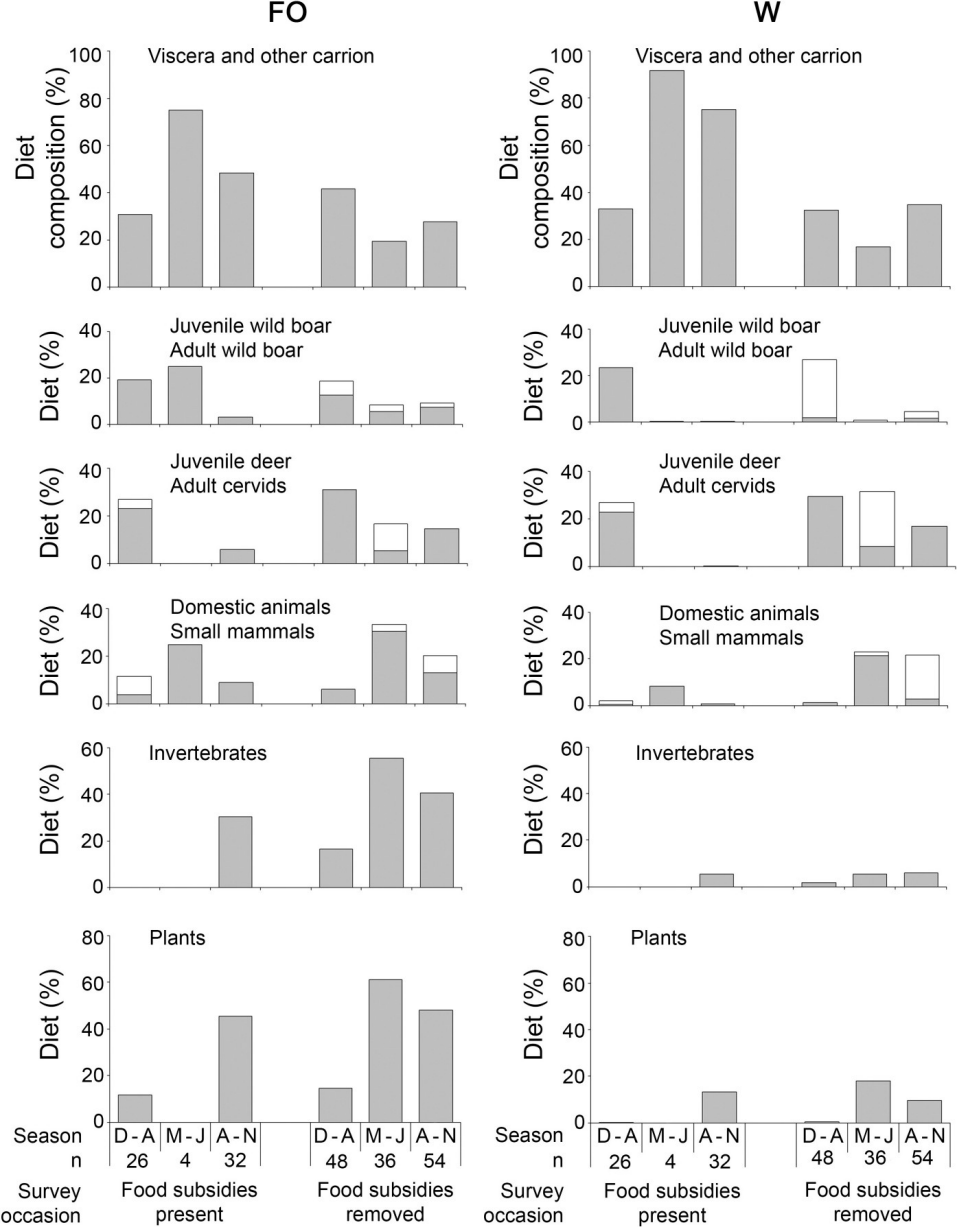
Seasonal stomach content composition of golden jackals in Hungary (Lábod region) depending on survey occasion (food subsidies present and food subsidies removed). FO–percentage frequency of occurrence, W–percentage weight of individual food remains found in the samples. n–number of samples.

In log-linear analysis the survey occasion was not a significant predictor of the consumption of any food types (Table 2). Compared to December-April, jackals consumed significantly more small mammals in May-July, and invertebrates and plants in May-November (Fig 4). Compared to males, females consumed more plants.

**Table 2:** Results of log-linear models for the frequencies of occurrence of food types in the stomachs of golden jackals in SW Hungary (Lábod region), for the effect of survey occasion (food subsidies present and food subsidies removed), seasons (December–April, May–July, August—November), sex and their interaction.

Item	Effect	df	χ^2^	P	Item	Effect	df	χ^2^	P
Viscera and	Survey occasion	1	2.40	0.1215	Brown hare	Survey occasion	1	0.38	0.5351
other carrion	Season	2	0.64	0.7257		Season	2	2.26	0.3224
	Sex	1	1.49	0.2229		Sex	1	1.02	0.3128
	Survey occasion × season	2	6.21	0.0448		Survey occasion × season	2	0.40	0.8197
	Survey occasion × sex	1	2.75	0.0975		Survey occasion × sex	1	0.33	0.5633
	Season × sex	2	0.09	0.9543		Season × sex	2	0.38	0.8264
Wild boar,	Survey occasion	1	0.88	0.3472	Domestic	Survey occasion	1	0.30	0.5860
adult	Season	2	3.34	0.1887	animals	Season	2	0.41	0.8153
	Sex	1	2.75	0.0973		Sex	1	1.10	0.2932
	Survey occasion × season	2	2.54	0.2813		Survey occasion × season	2	4.48	0.1067
	Survey occasion × sex	1	0.14	0.7097		Survey occasion × sex	1	0.01	0.9421
	Season × sex	2	0.47	0.7906		Season × sex	2	0.95	0.6222
Wild boar,	Survey occasion	1	0.06	0.8089	Birds	Survey occasion	1	0.18	0.6705
juvenile	Season	2	1.16	0.5593		Season	2	3.00	0.2236
	Sex	1	0.23	0.6309		Sex	1	1.18	0.2767
	Survey occasion × season	2	1.14	0.5644		Survey occasion × season	2	0.61	0.7383
	Survey occasion × sex	1	0.03	0.8699		Survey occasion × sex	1	0.10	0.7546
	Season × sex	2	1.44	0.4880		Season × sex	2	0.10	0.9535
Deer*,	Survey occasion	1	3.37	0.0665	Reptiles and	Survey occasion	1	0.99	0.3186
Adult	Season	2	5.79	0.0552	amphibians	Season	2	1.59	0.4520
	Sex	1	2.04	0.1530		Sex	1	0.00	0.9458
	Survey occasion × season	2	1.55	0.4617		Survey occasion × season	2	0.19	0.9107
	Survey occasion × sex	1	1.24	0.2660		Survey occasion × sex	1	0.35	0.5563
	Season × sex	2	5.13	0.0770		Season × sex	2	0.04	0.9805
Deer*,	Survey occasion	1	0.95	0.3303	Fish	Survey occasion	1	0.05	0.8286
juvenile	Season	2	7.28	0.0262		Season	2	1.50	0.4716
	Sex	1	0.18	0.6713		Sex	1	0.15	0.6984
	Survey occasion × season	2	0.16	0.9212		Survey occasion × season	2	3.21	0.2008
	Survey occasion × sex	1	0.51	0.4756		Survey occasion × sex	1	0.00	0.9915
	Season × sex	2	0.64	0.7275		Season × sex	2	0.12	0.9405
Roe deer,	Survey occasion	1	3.76	0.0526	Invertebrates	Survey occasion	1	5.56	0.0184
adult	Season	2	5.66	0.0591		Season	2	20.20	*0*.*0000*
	Sex	1	1.45	0.2279		Sex	1	0.15	0.7021
	Survey occasion × season	2	0.52	0.7727		Survey occasion × season	2	2.26	0.3224
	Survey occasion × sex	1	0.94	0.3324		Survey occasion × sex	1	0.03	0.8741
	Season × sex	2	1.16	0.5609		Season × sex	2	5.04	0.0806
Carnivores	Survey occasion	1	1.31	0.2518	Plants	Survey occasion	1	1.24	0.2653
	Season	2	0.28	0.8698		Season	2	24.79	*0*.*0000*
	Sex	1	0.34	0.5615		Sex	1	8.46	0.0036
	Survey occasion × season	2	1.38	0.5018		Survey occasion × season	2	2.95	0.2287
	Survey occasion × sex	1	0.00	0.9605		Survey occasion × sex	1	0.00	0.9644
	Season × sex	2	0.44	0.8023		Season × sex	2	1.38	0.5024
Small	Survey occasion	1	0.08	0.7754	Others	Survey occasion	1	0.38	0.5385
mammals	Season	2	12.60	*0*.*0018*		Season	2	1.81	0.4043
	Sex	1	2.52	0.1123		Sex	1	0.64	0.4251
	Survey occasion × season	2	0.12	0.9406		Survey occasion × season	2	3.54	0.1700
	Survey occasion × sex	1	0.05	0.8265		Survey occasion × sex	1	0.02	0.9007
	Season × sex	2	1.27	0.5308		Season × sex	2	1.77	0.4129

*Fallow deer (*Cervus dama*) or red deer (*Cervus elaphus*). Numbers in italics indicate significant values (P < 0.0031, Bonferroni correction).

In MANCOVA there was no significant difference in viscera and other carrion consumption either in the main effects (survey occasion, season, sex) or survey occasion × season interaction (Table 3). In the first survey occasion, jackals consumed a higher proportion of adult wild boar (W: 11.6% *vs*. 1.3%), while in the second survey occasion, juvenile wild boars (0 *vs*. 11.8%), domestic animals (0.8% *vs*. 6.2%) and invertebrates (2.6% *vs*. 4.1%) were more eaten (Fig 4). Compared to other seasons, jackals in December-April consumed significantly higher proportions adult wild boar while in August-November they consumed more domestic animals, invertebrates and plants. The survey occasion × season interaction was significant in some cases (Fig 4). Significantly more adult wild boar consumption occurred in the first survey occasion in December-April, while more domestic animal consumption occurred in the second survey occasion in August-November, and invertebrates were not detected during the first survey occasion in December-July. Compared with males, females consumed more plants (5.6% *vs*. 15.4%) (Fig 4).

**Table 3:** Results of MANCOVA for the wet weight of food types in the stomachs of golden jackals in SW Hungary (Lábod region), for the effect of survey occasion (food subsidies present and food subsidies removed), seasons (December—April, May—July, August—November), sex and survey occasion × season interaction.

Effect	Food categories	df	F	P	Effect	Food categories	df	F	P
Survey occasion	Viscera and carrion	1	3.89	0.106	Sex	Viscera and carrion	1	0.12	0.747
	Wild boar, adult	1	99.56	*0*.*000*		Wild boar, adult	1	0.28	0.618
	Wild boar, juvenile	1	10.81	*0*.*022*		Wild boar, juvenile	1	0.00	0.962
	Deer*, adult	1	2.92	0.148		Deer*, adult	1	0.53	0.500
	Deer*, juvenile	1	0.19	0.680		Deer*, juvenile	1	0.19	0.680
	Roe deer, adult	1	0.35	0.579		Roe deer, adult	1	0.40	0.556
	Carnivores	1	0.97	0.371		Carnivores	1	1.05	0.353
	Small mammals	1	0.09	0.773		Small mammals	1	2.27	0.192
	Brown hare	1	2.50	0.175		Brown hare	1	2.50	0.175
	Domestic animals	1	11.27	*0*.*020*		Domestic animals	1	4.98	0.076
	Birds	1	3.77	0.110		Birds	1	1.54	0.270
	Reptiles, amphibians	1	0.03	0.868		Reptiles, amphibians	1	0.09	0.775
	Fish	1	1.17	0.328		Fish	1	1.74	0.244
	Invertebrates	1	38.19	*0*.*002*		Invertebrates	1	2.91	0.149
	Plants	1	0.71	0.438		Plants	1	7.70	*0*.*039*
	Others	1	1.29	0.308		Others	1	1.46	0.281
Season	Viscera and carrion	2	0.57	0.599	Survey	Viscera and carrion	2	0.77	0.513
	Wild boar, adult	2	389.97	*0*.*000*	occasion	Wild boar, adult	2	308.12	*0*.*000*
	Wild boar, juvenile	2	2.40	0.186	×	Wild boar, juvenile	2	2.40	0.186
	Deer*, adult	2	0.80	0.501	season	Deer*, adult	2	0.00	0.998
	Deer*, juvenile	2	0.53	0.619		Deer*, juvenile	2	1.20	0.375
	Roe deer, adult	2	1.49	0.311		Roe deer, adult	2	0.35	0.719
	Carnivores	2	0.99	0.435		Carnivores	2	1.03	0.422
	Small mammals	2	3.27	0.124		Small mammals	2	0.00	0.997
	Brown hare	2	0.63	0.572		Brown hare	2	0.63	0.572
	Domestic animals	2	6.43	*0*.*042*		Domestic animals	2	12.16	*0*.*012*
	Birds	2	0.94	0.450		Birds	2	5.53	0.054
	Reptiles, amphibians	2	1.56	0.298		Reptiles, amphibians	2	0.08	0.928
	Fish	2	3.13	0.131		Fish	2	0.29	0.760
	Invertebrates	2	43.83	*0*.*001*		Invertebrates	2	14.11	*0*.*009*
	Plants	2	9.84	*0*.*018*		Plants	2	2.64	0.165
	Others	2	1.24	0.366		Others	2	1.49	0.311

*Fallow deer (*Cervus dama*) or red deer (*Cervus elaphus*). Numbers in italics indicate significant values (P < 0.05).

### Trophic niche and number of food items

Compared to the first survey occasion, the standardized trophic niche did not significantly differ between survey occasions for either RFO data (B_A_, mean ± SE, 0.25 ± 0.09 *vs*. 0.32 ± 0.05, paired samples t-test, t_2_ = 1.577, P = 0.256) and W data (0.10 ± 0.08 *vs*. 0.26 ± 0.03, t_2_ = 1.492, P = 0.274). Compared to the first survey occasion, the stomachs in the second survey occasion contained significantly more food items (mean ± SE, 1.79 ± 0.15 and 2.55 ± 0.15, independent samples t-test, t_174_ = 3.119, P = 0.002).

## Discussion

### Changes in food sources resulting from big game management

The removal of viscera did not result in a statistically significant decrease in its consumption. Despite the lack of statistical significance, the difference was biologically considerable, their consumption was nearly halved (frequency of occurrence: 45% *vs*. 30%; wet weight: 55% *vs*. 29%, respectively). There could be several explanations for this. Firstly, the annual pattern of viscera and carrion left during intensive big game management are related to the characteristics of hunting practices, e.g. to legal hunting seasons [15, 43], and injured ungulates and carcasses from other mortality causes in this area. Although there were differences between surveys in viscera availability, these anthropogenic food subsidies are available in the highest quantities for scavengers (including the jackal) in autumn and winter (Figs 1 and 2). In these otherwise critical periods, the scattered and easily available foods with high energy values help animals to survive. For example, fat deposited in autumn can helps overwintering medium-sized canids, e.g. foxes [44] or coyotes [45]. During the winter, the amount of available food is relatively scarce [17, 46] without anthropogenic food subsidies (S1 Film). With these, as in our study area, jackals were the heaviest in the December-April period, which is also the mating season of the jackal [40], which is associated with more intense daily and territorial activity [11, 21, 31] and therefore greater energy requirements. Although the amount of the big game viscera drastically declined in spring and summer (period of pupping or calving and early parental care), carcasses were still available in large numbers during this period (it was impossible to remove them all). So in spring and summer the importance of viscera reduction is small.

Secondly, the professional hunters could not remove all viscera from the area. Outside the study area viscera was accessible and we cannot rule out that some of the 44 jackal groups we recorded in our study area (see Material and methods) might have immigrated from beyond the area where viscera were not removed. The extent of the study area (besides the relatively high jackal density) was enough big to reduce the occurrence of examining animals from outer areas. Poaching with snares and guns is common in the region [11, 47]. Besides large quantities of fresh deer meat, a piece of a leather glove [11] and a bullet from an illegally used gun were found within jackal stomachs, indicating presence of poaching. Therefore wounded individuals and remains of ungulates still occurred in the area despite our efforts to remove them. Viscera eating by jackals from these individuals is also not derived from direct predation or predation on healthy ungulates, that is, the cleaning role of jackals [15] is more decisive. Wounding (from hunting and poaching) and vehicle collisions leave big game carcasses throughout the year [48], and some of these are not found (unregistered). Therefore, the amount of big game carrion is presumably underestimated in the area.

Thirdly, golden jackals are socially flexible and neighbouring groups are able to reduce their normal territorial antagonism and share locally abundant food sources [8, 17]. The big game carcasses contribute to the increased need for food during the pup rearing period [29–30]. There was no significant difference between survey occasions in the quantity of registered carcasses.

Fourthly, a part of the consumed viscera may have been derived from carcasses. In intensive big game management areas, where ungulates are available from many sources, they are very important food resources for jackals [11, 49], alongside domestic animals and garbage [50–51]. Throughout the year (not just during calving), jackals can find a large variety of big game species.

### Changes in stomach content weight and body mass

Contrary to our first prediction, food removal did not significantly increase the proportion of empty stomachs and did not significantly reduce stomach weight. The low percentage (10–13%) of empty stomachs was similar (14–15% [43, 52]) or smaller (20–24% [48, 53]) to other studies. This indicates that the available food sources were high, although stomach content weight was lower than others [15] found in winter (190 g). Because we found that stomach content weight in December-April of the second survey occasion was significantly lower than in the first, it seems that during the critical winter-early spring period [46], a decrease in food intake can occur. Overall, the food supply has remained favourable for jackals despite the reduction in anthropogenic food subsidies.

The body mass analysis only partially supported the first prediction that big game viscera removal results in reduced body mass. We observed significant effects only in the survey occasion × sex interaction. The different effect on each sex may be explained by the burden associated with pregnancy and lactation in females compared to males [29–30]. Therefore the negative effect associated with viscera removal is likely to affect females more, so it could lower the body mass. In addition, females consume a higher proportion of less nutritious plants [54], which may also have contributed to their lower body mass.

### Intraspecific differences in diet

Contrary to our hypothesis, the primary food of the jackals remained viscera and carrion of big game despite their reduced availability. This is related to the changes in food sources resulting from big game management. The regression analysis showed no strong relationship between the consumption of big game and the availability of viscera and carrion. That is, with low big game viscera and carcass availability, consumption of big game can still be considerable. We collected data from acoustic surveys to explore the numerical responses of the jackal population to big game viscera removal, but observed only a low decrease in family group density and increase in single jackal density. Furthermore, in the second survey, reproduction among one-year old females was also observed. Presumably, food reduction in less productive areas [5, 21] compared to areas of high ungulate density can result in greater impacts of decreasing population density and survival, and increasing home range size of medium-sized canids. To better understand the ecology of the jackal, during a long-term period, for example population size, reproduction and habitat use, parallel with feeding habits should be analysed in relation to food abundance (or: amount of food available).

Overall, even the seemingly small amount of anthropogenic food subsidies in our high ungulate density area is sufficient to sustain the jackal population, as well as other species that rely on scavenging, such as wild boar, common ravens (*Corvus corax*) and white-tailed eagles (*Haliaeetus albicilla*). Similarly, no relationship was found between small mammal availability and small mammal consumption by the jackal and the red fox in Hungarian agroecosystems [23]. Therefore, in addition to rodent control, our study illustrates the sanitary or cleaning role of jackals [11, 15]. In addition to the above mentioned problematic issues of viscera removal and access to carcasses (e.g. changes in quantity within the year, poaching, wounding and vehicle collision), data collection (hunting bag data, carrion registration; [55]) and investigation methodological constraints [56] could have contributed. From stomach analysis, it is often impossible to separate viscera consumption and eating from carrion [11, 43]. Because it was only viscera removal, but no carrion removal (it was not feasible), the use of the combined food category (viscera and other carrion) may mask a part of the actual impact of the food manipulation.

The feeding responses of the jackal to the source reduction were less pronounced than expected in the case of other food types. Contrary to our second prediction, we did not find significant differences between treatment periods in log-linear analysis of any of the main food types. However, with MANCOVA, we found treatment period differences in consumption of some food types, but, many other (presumed) food types (e.g. small mammals, young cervids), had no statistically significant increase in consumption ratios. However, less adult wild boar were consumed in the second survey occasion. The consumption of adult wild boar by a mesocarnivore is more likely to be caused by scavenging, than predation [43, 52], although the predation e.g. on wounded, sick individuals cannot be excluded. Wild boar population densities depend on the severity of winter [57], however our study site experienced no major differences in weather conditions (S1 Table) and wild boar population (S2 Table) between the study periods. Increased consumption of young wild boars indicates food shift, which supported our prediction. Wild boar young are close to the 4–5 kg preferred weight category of the golden jackal [58] for hunting. Food switching by mesocarnivores from scavenging to predation on young of wild ungulates has been observed in the case of high scavenger or predator abundance [9, 30, 36, 59]. As we have assumed, due to the removal of viscera, jackals consumed more food from garbage or dumps (indicated by inorganic materials and domestic animals, Table 1), however we detected lower consumption rates from garbage than in southern Europe [15, 43, 51–52]. This, alongside the high wild ungulate abundances, can be related to the low human population density in the study area, while jackal home ranges are also affected by settlements [6, 31].

### Management implications

In the absence of large carnivores (top-down regulation), the abundance of mesopredators is usually limited by available food resources [12, 60], and bottom-up regulation prevails. Food abundance has an influence on coyote (*Canis latrans*) numbers, reproductive rates, survival, dispersal and space-use patterns [61], and this has been demonstrated experimentally on red fox [5] and golden jackal [21]. Leaving big game viscera or of domestic animal carcasses and garbage [15, 43, 49], can maintain the population of scavengers [5, 9, 21, 24, 62]. For this reason, the effect of even an enforced resource reduction may be moderate.

In conclusion, the feeding responses of jackal to the reduction of food subsidies were less pronounced than expected despite 50 kg of viscera removed per km per year. Because in high big game density areas, wild ungulate carrion from different mortality causes are available in high quantities throughout the year, predator populations can be maintained despite the high amount of viscera removal.

## Supporting information

S1 Fig Geographic locality, main habitat types and sampling site of the study area (Lábod region, SW Hungary). (TIF)

S1 Table Meteorological data of the study area (Lábod region, SW Hungary). Source of climate data: Hungarian Meteorological Service.
(DOC)

S2 Table Harvest density (individuals/km^2^) of game species in Lábod region (SW Hungary). Source: Hungarian Game Management Database (http://ova.info.hu).
(DOC)

S1 Film Wild-living adult golden jackal (*Canis aureus*) eating the viscera of big game. Jackals eat considerable amounts of meat quickly, in relatively large chunks. The 58-second film was made by Zoltán Horváth (Danube-Drava National Park Directorate) in daylight, ca. 15 kilometres away from our study area.
(MP4)
