# Supplementary figures and images for: Feeding responses of the golden jackal after reduction of anthropogenic food subsidies
Source: PLoS One. 2018 Dec 7;13(12):e0208727. doi: 10.1371/journal.pone.0208727 (PMC6286136; doi:10.1371/journal.pone.0208727)

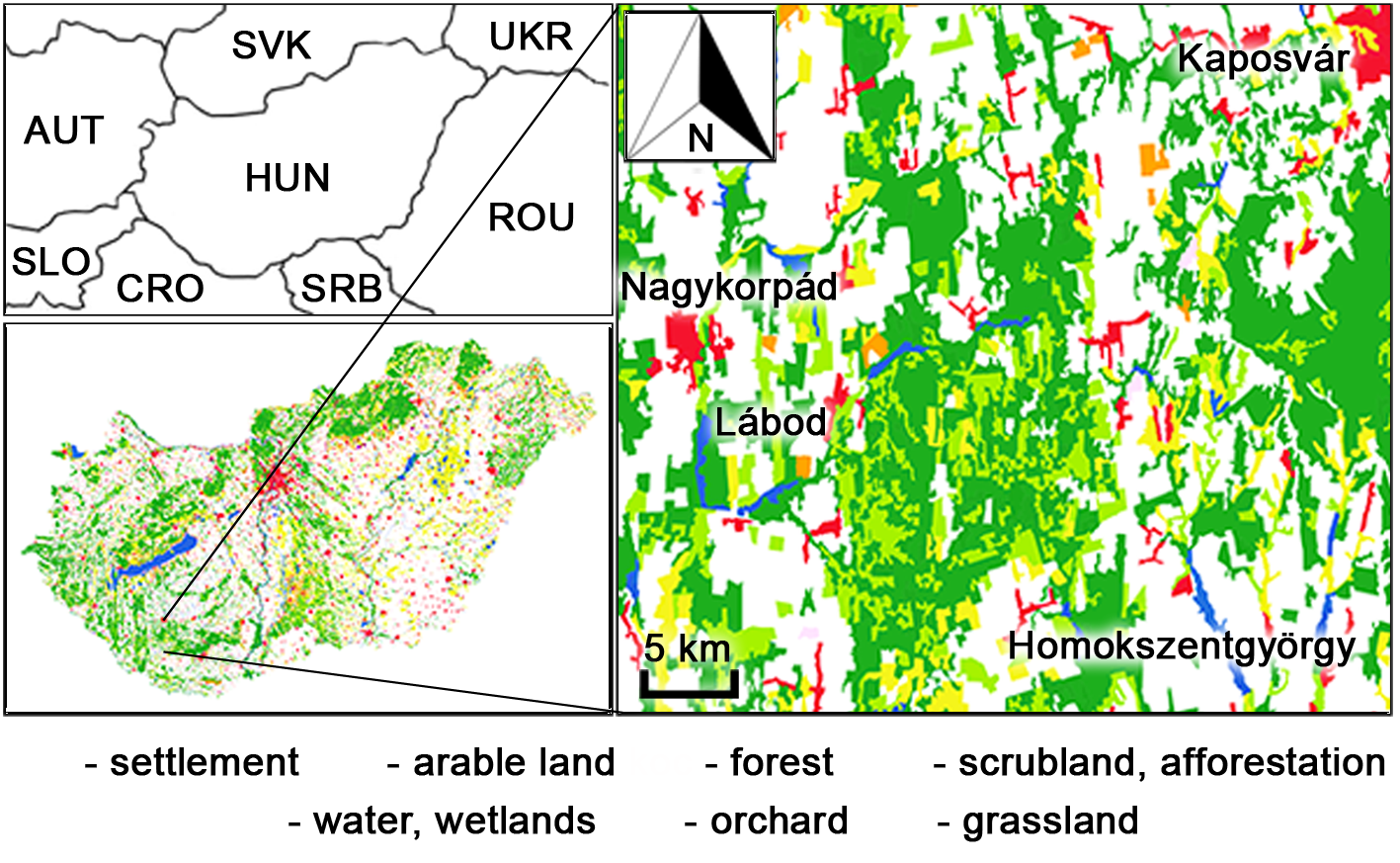

Supplement: S1 Fig — (TIF) [file pone.0208727.s001.tif]
