# Supplementary material for: Feeding responses of the golden jackal after reduction of anthropogenic food subsidies
Source: PLoS One. 2018 Dec 7;13(12):e0208727. doi: 10.1371/journal.pone.0208727 (PMC6286136; doi:10.1371/journal.pone.0208727)
Supplement: S1 Table — Source of climate data: Hungarian Meteorological Service. (DOC) [file pone.0208727.s002.doc]

S1 Table.

| Year | Number of | | Number of | | Average | | Mean temperature (°C) | | | | | | Annual |
| --- | --- | --- | --- | --- | --- | --- | --- | --- | --- | --- | --- | --- | --- |
|  | frost days | | days with | | snow | |  | |  | |  | | precipitation |
|  |  | | snow cover | | depth (cm) | | Winter | | Summer | | Annual | | (mm) |
| 2011 | 115 | 18 | | 0.8 | | 0.0 | | 20.2 | | 10.3 | | 456 | |
| 2012 | 85 | 13 | | 3.0 | | 0.3 | | 21.4 | | 11.3 | | 526 | |
| 2013 | 82 | 35 | | 2.5 | | 1.2 | | 20.3 | | 10.8 | | 876 | |
| 2014 | 53 | 3 | | 2.0 | | 3.3 | | 19.4 | | 11.6 | | 1011 | |
| 2015 | 73 | 14 | | 1.5 | | 2.4 | | 21.4 | | 11.5 | | 693 | |
