# Supplementary material for: Feeding responses of the golden jackal after reduction of anthropogenic food subsidies
Source: PLoS One. 2018 Dec 7;13(12):e0208727. doi: 10.1371/journal.pone.0208727 (PMC6286136; doi:10.1371/journal.pone.0208727)
Supplement: S2 Table — Source: Hungarian Game Management Database (http://ova.info.hu). (DOC) [file pone.0208727.s003.doc]

S2 Table.

| Year | Red | Fallow | Roe | Wild | Golden | Red |
| --- | --- | --- | --- | --- | --- | --- |
|  | deer | deer | deer | boar | jackal | fox |
|  | Hunting bag (individuals/km^2^) | | | | | |
| 2010/2011 | 1.31 | 3.36 | 0.38 | 1.18 | 0.05 | 0.12 |
| 2011/2012 | 1.30 | 3.24 | 0.38 | 1.64 | 0.13 | 0.11 |
| 2012/2013 | 1.04 | 1.88 | 0.32 | 2.83 | 0.20 | 0.11 |
| 2013/2014 | 1.02 | 1.67 | 0.30 | 1.82 | 0.20 | 0.12 |
| 2014/2015 | 0.90 | 1.34 | 0.18 | 2.21 | 0.29 | 0.10 |
| 2015/2016 | 1.02 | 1.20 | 0.22 | 2.39 | 0.27 | 0.10 |
| Mean | 1.10 | 2.12 | 0.30 | 2.01 | 0.19 | 0.11 |
| ± SE | 0.07 | 0.39 | 0.03 | 0.24 | 0.04 | 0.01 |
